# Intracranial hypertension due to spinal cord tumor misdiagnosed as pseudotumor cerebri syndrome: case report

**DOI:** 10.1186/s12883-020-02000-y

**Published:** 2020-11-19

**Authors:** Wanglu Hu, Chun Wang, Qun Wu, Yike Chen, Wei Gao, Guangyu Ying, Yongjian Zhu, Wei Yan

**Affiliations:** Department of Neurosurgery, the Second Affiliated Hospital, Zhejiang University School of Medicine, 88 Jiefang Rd, Hangzhou, 310009 China

**Keywords:** Intracranial hypertension, Pseudotumor cerebri syndrome, Spinal tumor, Schwannoma, Venous sinus stenosis

## Abstract

**Background:**

Isolated onset of intracranial hypertension due to spinal cord tumor is rare, thus, easily leading to misdiagnosis and delay in effective treatment.

**Case presentation:**

Herein, we describe a 45-year-old female patient who manifested isolated symptoms and signs of intracranial hypertension and whose condition was initially diagnosed as idiopathic intracranial hypertension and transverse sinus stenosis. The patient received a stent implantation; however, no improvements were observed. One year later her symptoms exacerbated, and during rehospitalization a spinal imaging examination revealed a lumbar tumor. Pathologic evaluation confirmed schwannoma, and tumor resection significantly improved her symptoms, except for poor vision.

**Conclusions:**

Space-occupying lesions of the spine should be considered in the differential diagnosis of idiopathic intracranial hypertension, even in the absence of spine-localized signs or symptoms.

## Background

Intracranial hypertension (ICH) secondary to spinal cord tumor is a relatively rare, but well-described manifestation. With a concomitant diagnostic ratio of 47% [1], its diagnosis is not particularly difficult when typical spinal symptoms or signs are present. However, absence of spinal cord signs could lead to misdiagnosis of idiopathic intracranial hypertension (IIH), also known as pseudotumor cerebri syndrome, which is defined as ICH with unknown etiology. Once misdiagnosed, a delay in treatment or unnecessary treatments can result in severe consequences for patients.

We describe a patient who manifested isolated symptoms and signs of ICH. Her condition was initially misdiagnosed as IIH and venous sinus stenosis. However, one year later a confirmed diagnosis of lumbar schwannoma was made. We also review the literature describing previous similar cases and share our views on associated pathophysiology and therapies.

## Case presentation

A 45-year-old female patient presented with blurred vision for 13 months and was initially diagnosed with papilledema at the ophthalmology department of another hospital. With no effective treatment, her condition deteriorated over the next 2 months. Consequently, she was admitted to the neurology department of our hospital. No other symptoms of ICH (headache, nausea, vomiting) or any other neurologic deficits were present on admission. The medium-build patient had no previous medical history or family history of venous thromboembolism or hematological diseases, and she denied any drug history including the use of oral contraceptive pills.

**Fig. 1:**
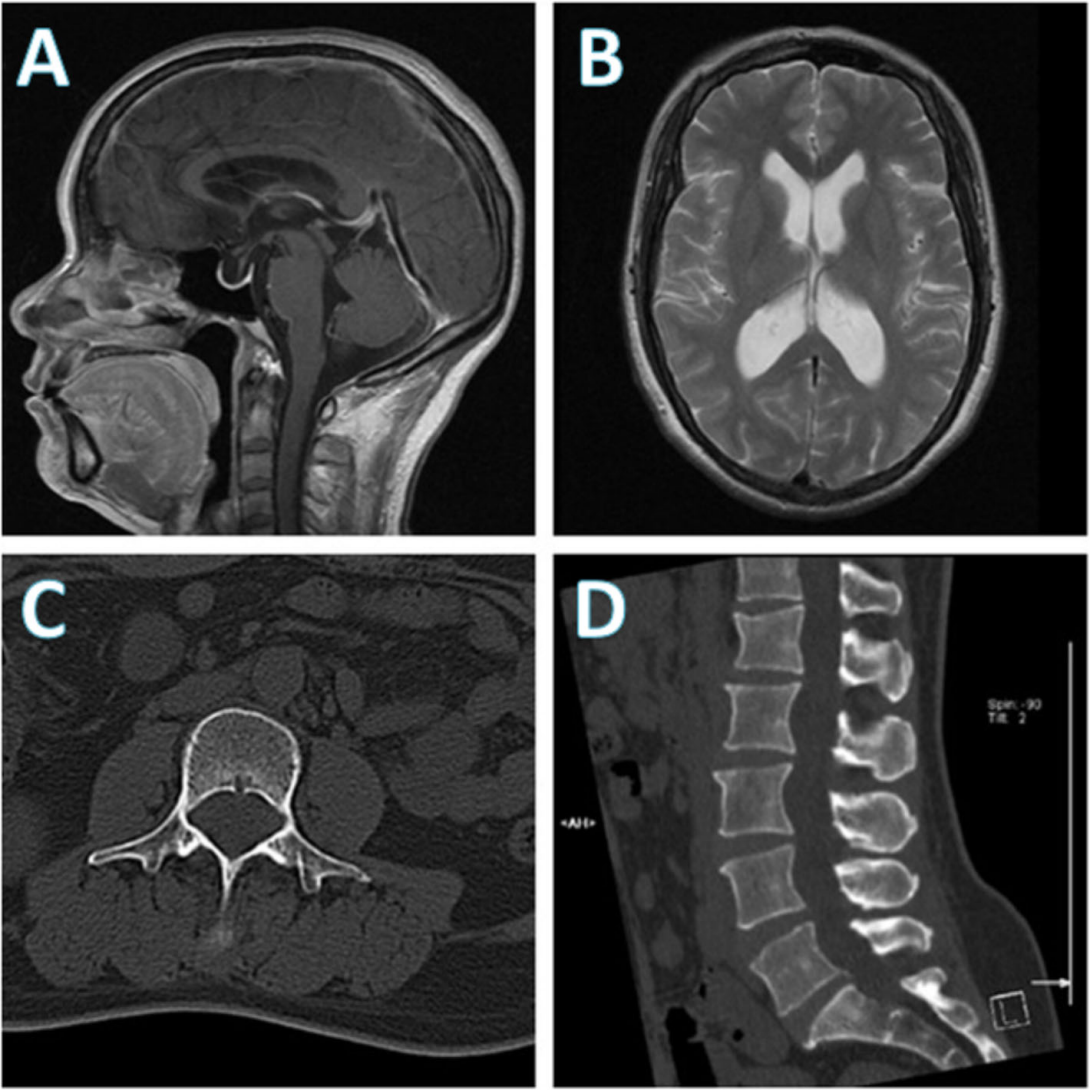
Brain magnetic resonance imaging (MRI) and lumbar computed tomography (CT) performed during the patient’s first admission. **a** Brain MRI demonstrated mild enlargement of the supratentorial ventricle, **b** the abnormal sign of vacuolar sella in the right optic-radiation of lateral thalamus, bilateral medial temporal lobes, and the insular lobes. **c**, **d** No abnormalities were found in lumbar CT at first; however, a retrospective review of the spinal CT scan (**d**) showed the evidence of enlarged neural foramina and mild vertebral scalloping which suggested a long-standing intradural tumor such as schwannoma

Physical examination revealed a slower light reflex of the left eye, decreased visual acuity of bilateral eyes (more severe on the left) and left nasal hemianopsia. On neurological examination, no neurological localizing signs were observed. Fundus examination showed bilateral papilledema. Brain magnetic resonance imaging (MRI) showed no occupying lesions, only mildly dilated ventricles (Fig. 1a and b). Lumbar puncture (LP) revealed a significantly elevated pressure of 330 mmH_2_O. Laboratory examination of cerebrospinal fluid (CSF) indicated a normal cell count of 4 × 10^6^/L (normal range 0–8 × 10^6^/L) and a significantly increased protein level of 382.6 mg/dL (normal range 0–43 mg/dL). Given the other negative results of CSF ink stain, blood T-SPOT tuberculosis, and normal computed tomography (CT) scan of the lung, the neurologists excluded intracranial tuberculous and cryptococcal infection. Meningeal carcinomatosis was also not considered due to the slow progression, though CSF cytology was not investigated.

**Fig. 2:**
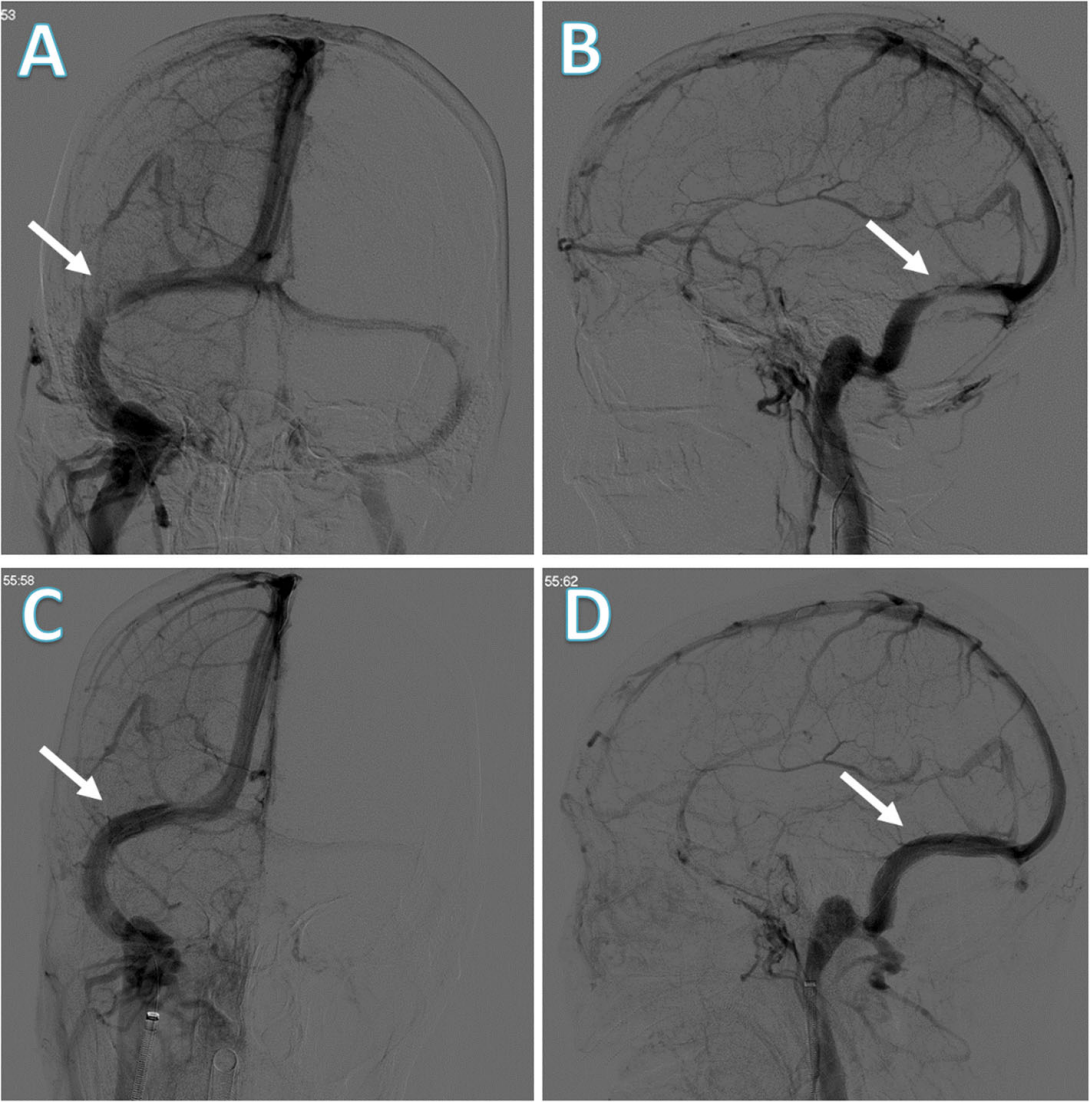
Brain digital subtraction angiography (DSA) before and after stent implantation. **a**, **b** The pre-implantation DSA suggested an obvious stenosis (arrows) localized in the area of the right transverse sinus. **c**, **d** Post-implantation DSA showed the well-dilated stent was in position and venography identified a smooth venous reflux (where the arrow points to)

A diagnosis of cranial venous sinus thrombosis (CVST) was then suspected and contrast-enhanced magnetic resonance venography (CE-MRV) was performed. The examination showed relatively thin transverse sinus on both sides but no obvious evidence of CVST. Considering the possibility of elevated CSF protein caused by extracranial, and not intracranial, tumors, the neurologists planned for lumbar MRI but failed to perform the examination because of a metal intrauterine device (IUD). Instead, a lumbar CT scan was performed, revealing no abnormalities (Fig. 1c and d). Still, evidence supporting secondary ICH was insufficient. Hence, a digital subtraction angiography (DSA) was performed which showed an obvious stenosis localized in the area of the right transverse sinus, and the venous pressure gradient near the stenosis reached 28 mmHg, which confirmed venous sinus stenosis (Fig. 2a and b). She was then diagnosed with IIH and transverse sinus stenosis (TSS).

A few days later, the patient received a percutaneous intracranial right transverse sinus stent implantation. DSA showed that the well-dilated stent was in position, and venography identified a smooth venous reflux (Fig. 2c and d). However, her symptoms did not improve. Two days later, a repeat LP revealed that the pressure was still high at 305 mmH_2_O. Further examinations were suggested but the patient refused and was discharged.

**Fig. 3:**
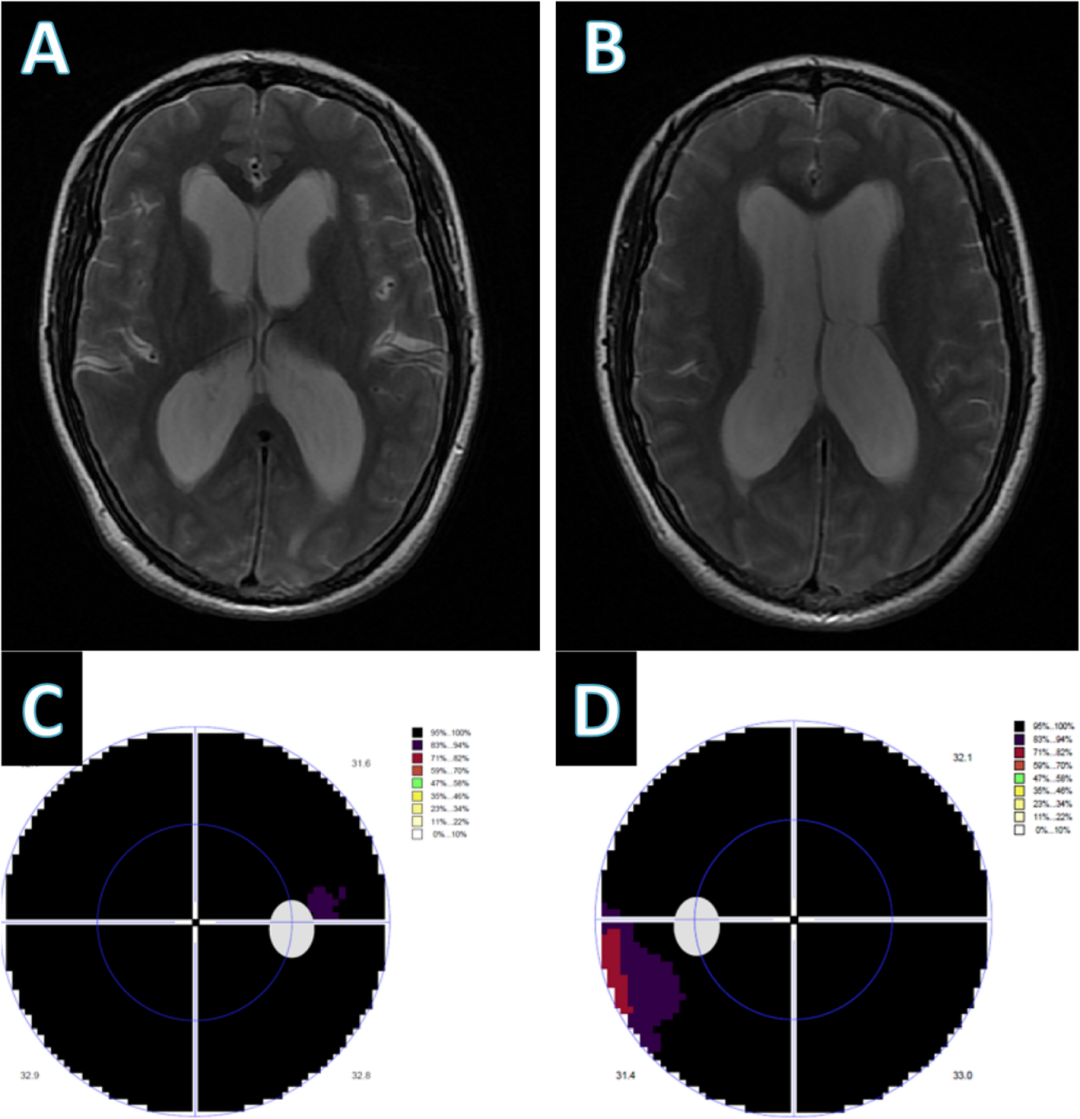
Brain magnetic resonance imaging (MRI) and visual field examination during the second admission 1 year later. **a**, **b** Brain MRI showed more pronounced hydrocephalus than before. **c**, **d** Ophthalmologic examinations revealed almost complete loss in bilateral visual fields

**Fig. 4:**
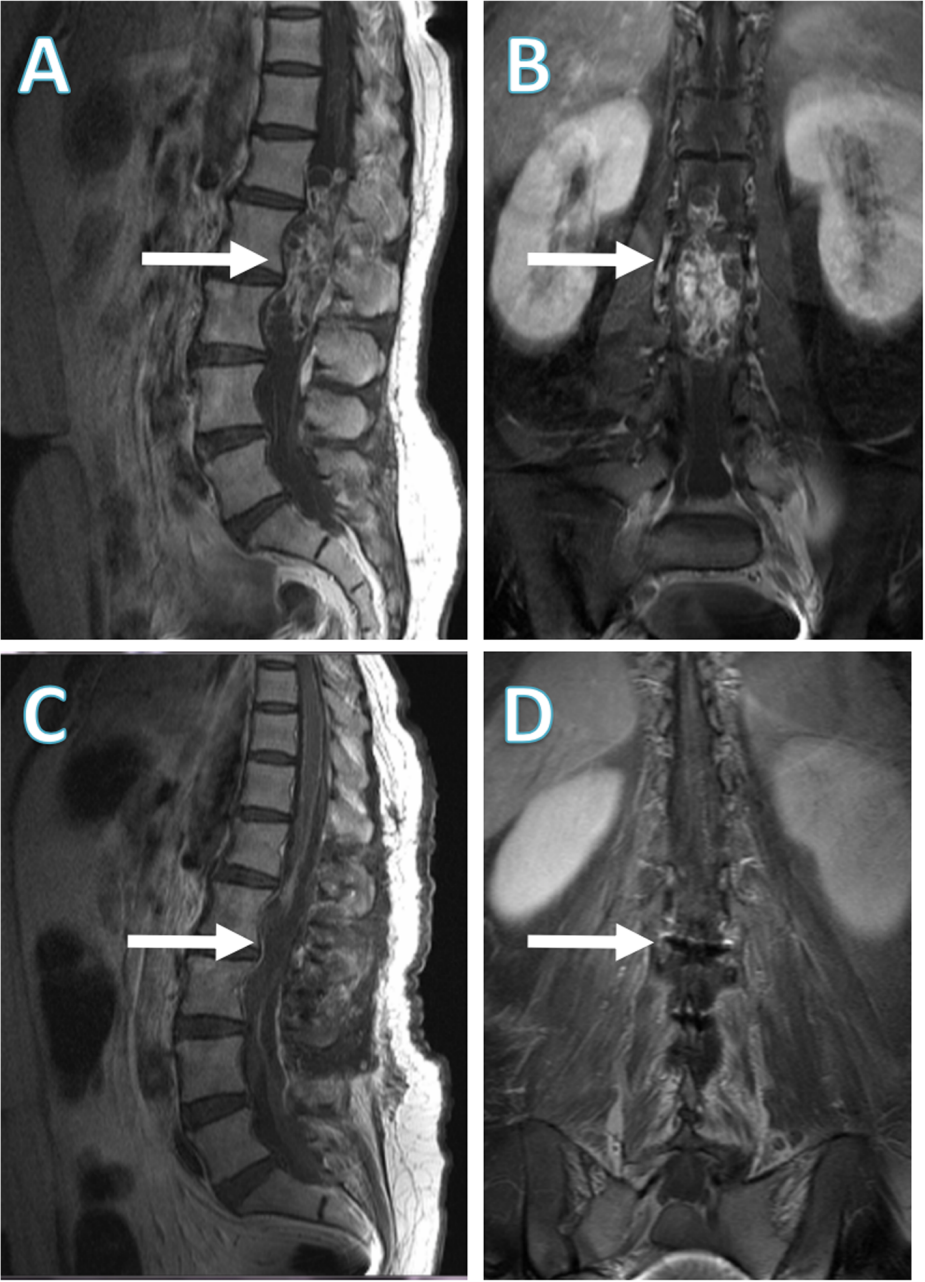
Lumber magnetic resonance imaging (MRI) examinations before and after tumor resection. **a**, **b** The preoperative lumbar MRI indicated an occupying lesion at the 1–3 lumbar vertebrae (arrow). **c**, **d** The postoperative lumbar MRI indicated a complete resection of the tumor (arrow)

One year later, the patient returned to our hospital due to an exacerbated condition. Her vision had worsened and she had been experiencing severe headaches accompanied by frequent nausea and vomiting for over 1 month. A brain MRI revealed obvious hydrocephalus, which was much more serious than that 1 year ago (Fig. 3a and b). She was admitted to our department and was scheduled to receive shunt surgery. Physical examination showed much slower light reflex of the left eye and worsening visual acuity than before. No neurological localizing signs were found on neurological examination. Ophthalmological examinations revealed severe papilledema and deficits in bilateral visual fields (Fig. 3c and d). Surprisingly, an LP performed the next day revealed an opening pressure of 320 mmH_2_O with fast-flow leakage of CSF at first, which slowed to no leakage within a short time. Repeated adjustment of the puncture needle was not successful and the Queckenstedt-Stookey test was positive. The CSF test showed a significantly increased protein level of 384.5 mg/dL. By then we highly suspected an intraspinal obstruction, or more precisely, a spinal tumor. Since the patient’s metal IUD had been taken out, a total spinal MRI examination was soon arranged. Imaging indicated an intramedullary tumor at the L1–L3 level and no abnormalities in the cervical or thoracic vertebrae (Fig. 4a and b).

One week after admission, the patient underwent resection of the L1–L3 tumor (Fig. 4c and d). The surgery was successful and pathologic evaluation confirmed schwannoma. On the first postoperative day, her headache was prominently relieved. However, her poor vision was not resolved, which may be caused by optic atrophy due to long-term suppression of ICH. A postoperative head CT scan showed a smaller ventricle than that observed in preoperative imaging. After approximately 2 weeks of recovery, the patient was discharged. A telephone follow-up 1 year later revealed that there had been no further deterioration in her symptoms, though her vision had still not recovered.

## Discussion and conclusions

Compared with typical neurological symptoms due to spinal lesions, such as backache, radiculalgia, sphincter dysfunction, and peripheral neurologic deficits, ICH and hydrocephalus secondary to intraspinal tumors are well-known but rare. According to a study published in 2004 [1] reporting 269 cases with rare complications, hydrocephalus occurred long before the onset of primary symptoms related to the spinal cord in approximately 29% of cases. Notably, the uniqueness of the current case is that the patient was once misdiagnosed as having IIH, also known as pseudotumor cerebri syndrome. Patients with this disorder can be classified into two subgroups: individuals with unclear causes, also known as IIH, and individuals with identifiable secondary causes. A definite diagnosis of IIH should fulfill the revised diagnostic criteria proposed in 2013 [2]. According to strict diagnostic procedures, misdiagnosing patients as having IIH is somewhat inevitable if no other neurological deficits appear, apart from isolated symptoms or signs of ICH.

We reviewed the literature on cases of spinal tumors that were initially misdiagnosed as IIH with absence of spinal symptoms or signs and found three similar cases (Table 1). Ahmed et al. reported two cases with spinal lymphoma [3]. One involved a 49-year-old man who complained of blurred vision and headache. Similar to our case, he had venous stenosis in the posterior sagittal and right transverse sinus. He received a stent implantation, an optic nerve sheath fenestration, and a ventriculoperitoneal shunt to relieve his symptoms. However, a later biopsy confirmed lymphoma. The symptoms of ICH improved after chemotherapy, but his vision was permanently impaired. In another similar case, the patient died 4 months after chemotherapy. Porter et al. reported a patient with initial presentations of bilateral papilledema and blurred vision who was considered to have IIH at first [4]. Not until his spinal cord symptoms developed was the diagnosis confirmed to be spinal astrocytoma. Eventually the patient became permanently paraplegic. Returning to the present case, the significantly increased CSF protein level and the mildly enlarged ventricles failed to meet the diagnostic criteria of IIH and were highly indicative of a secondary pathogenesis. It was unfortunate that the patient was contraindicated to MRI examination, on her first admission, because of the metal IUD. Even so, in retrospect, we should always keep in mind that in clinical practice once a secondary cause of ICH is suspected, further necessary examinations are urgently required. In this case, persuading the patient to remove the IUD earlier or performing the MRI at a lower magnetic field intensity could have benefited her prognosis. The above cases indicate that isolated initial manifestation of ICH with lack of localizing signs in the spinal cord could lead to misdiagnosis and delayed surgical intervention, which ultimately, could result in disastrous consequences. Clinicians need a broader differential spectrum of ICH for accurate and timely diagnosis.

**Table 1:** Reported cases of spinal cord tumor manifesting isolated signs and symptoms of intracranial hypertension

Case	Age/Sex	Presenting signs and symptoms	Spinal symptoms	Pathology	Treatment after misdiagnosis	Outcome
Ahmed et al. [3]	43/F	Headache, blurred vision, bilateral papilledema	None	Lymphoma	Lumbar puncture	Died 4 months after diagnosis
Ahmed et al. [3]	49/M	Headache, bilateral Papilledema, hearing loss	None	Lymphoma	Venous sinus stenting, Optic nerve sheath fenestration	A little better than before
Porter et al. [4]	19/M	Blurred vision	After lumboperitoneal shunt	Astrocytoma	Lumboperitoneal shunt	Completely paraplegia

Interestingly, our patient was misdiagnosed as having IIH accompanied by TSS. TSS generally represents a common sign of ICH [5, 6]. Early in 1995, King et al. discovered an elevation in cranial venous pressure in patients with IIH, which led to a growing recognition that local venous obstruction played an important role in the pathogenesis of IIH [7]. Later, other studies reported similar findings [8, 9]. Given the strong evidence gathered over the past two decades, venous sinus stenosis, in particular, TSS, has long been viewed as a contributor to the pathophysiology of IIH [10]. It is postulated that in patients with IIH, venous sinus stenosis and a subsequent increase in venous pressure initially appear to be the downstream consequence of elevated CSF pressure. The mild rise in intracranial pressure would in turn reflect on the venous compression and extrinsic stenosis, leading to a persistent elevation in pressure gradient between the cerebral venous sinus stenosis and subarachnoid space. This would result in decreased CSF absorption into the superior sagittal sinus and reduced drainage through the arachnoid granulations, thereby, further elevating intracranial pressure and perpetuating the positive feedback [11–14]. Retrospectively, it is more likely that the stenosis over the junction area of the right transverse sinus in our patient was the result of ICH rather than the reason for it. Endovascular stenting implantation in the stenosis is considered safe and effective for treating patients with venous hypertension with a stenosis and pressure gradient [14]; however, in our patient, no response was observed after stent implantation, which also indicated a secondary cause of ICH.

It is worth mentioning that the Queckenstedt-Stookey test plays a critical role in the final diagnosis. If the Queckenstedt-Stookey test would have been performed at her first admission, it might have been possible to diagnose the underlying lumbar tumor earlier, so as to improve her deteriorating vision. Hence, the present case also highlights the importance of an overall clinical examination in clinical practice.

As yet, the pathophysiological mechanism underlying the association between spinal tumors and ICH is not well established. Prevailing hypotheses include intraspinal neoplastic suppression, intracranial metastasis, increased CSF viscosity, increased CSF fibrinogen, reduced CSF compliance, and neoplastic arachnoiditis. Almost any type of spinal lesion can manifest signs of ICH. The relationship between ICH and cervical tumors can be explained. Any obstruction in the upper spinal canal could result in a rise in venous pressure and subsequent ICH, because venous reflux of the upper trunk mainly proceeds in the superior vena cava that localizes near the third thoracic vertebra. However, interpreting the pathophysiology in lower spinal tumors is not so easy and led us to question the present case manifesting as a lumbar schwannoma. A possible explanation regarding CSF dynamics is that a spinal obstruction could compromise lumbosacral region compliance, which is also known as an “elastic reservoir” for CSF flow, thereby isolating the spinal subarachnoid space from the intracranial region and impacting normal CSF compensation due to pressure alterations [1]. The findings of Morandi et al. in cases of benign spinal cord tumor support this theory [15].

Furthermore, a significant increase in CSF protein is a common sign in these patients. Several researchers have correlated the increased CSF protein with increased CSF viscosity, which would in turn increase the resistance to CSF absorption by arachnoid villi [4, 16, 17]. However, many studies disagree with this theory, because high CSF protein levels do not occur in all cases and, in fact, they have little effect on CSF viscosity [1, 18]. Other investigations have also reported that the increased CSF fibrinogen would suppress the absorption of CSF and cause ICH. The transformation of CSF fibrinogen into fibrin in the subarachnoid space and villi may increase the resistance to CSF outflow. The abnormal presence of fibrinogen may result from chronic inflammation, breakdown of the blood-brain barrier, or subarachnoid hemorrhage [19]. Furthermore, direct secretion from the neoplasm or a meningeal reaction to the neoplasm can both increase CSF protein [4]. In our case, no intracranial lesion was found and the tumor was located inside the spinal cord in the lumbar segment. Therefore, the evidence of neoplastic compression and intracranial metastasis causing cranial venous hypertension was inadequate for the present case. Considering the prominently lowered postoperative CSF pressure and protein level, as well as the improved symptoms, the potential mechanism underlying our case may be due to decreased CSF absorption. We suppose that increased CSF protein promotes elevation of CSF fibrinogen as well as CSF viscosity. Two pathological factors block the pathway of CSF reflux, which travels from a semipermeable membrane of the arachnoid granulation to the superior sagittal sinus, thus reducing CSF absorption and leading to an increase in intracranial pressure.

For treatment of such cases, resection of the primary spinal lesion is the first choice. Many patients can benefit from this surgery, even though some may have refractory hydrocephalus and may need to undergo shunt surgery. Moreover, if shunt surgery is performed first, there is a risk of carcinomatous spread in malignant cases.

In conclusion, this rare case highlights that clinicians should always maintain a broad differential spectrum of Idiopathic intracranial hypertension, even with the absence of intracranial lesions or neurologic deficits. Abnormal CSF compositions and imaging signs of hydrocephalus in cases with ICH may indicate spinal pathology. Misdiagnosis might result in unnecessary treatments, or more importantly, lead to permanent damage in patients.

## Data Availability

The datasets used and analyzed during the current study are available from the corresponding author on reasonable request.

## Abbreviations

CSF: Cerebrospinal fluid
CT: Computed tomography
DSA: Digital subtraction angiography
ICH: Intracranial hypertension
IIH: Idiopathic intracranial hypertension
MRI: Magnetic resonance imaging
TSS: Transverse sinus stenosis
